# Identification of Clostridioides difficile-Inhibiting Gut Commensals Using Culturomics, Phenotyping, and Combinatorial Community Assembly

**DOI:** 10.1128/mSystems.00620-19

**Published:** 2020-02-04

**Authors:** Sudeep Ghimire, Chayan Roy, Supapit Wongkuna, Linto Antony, Abhijit Maji, Mitchel Chan Keena, Andrew Foley, Joy Scaria

**Affiliations:** a Department of Veterinary and Biomedical Sciences, South Dakota State University, Brookings, South Dakota, USA; b South Dakota Center for Biologics Research and Commercialization, Brookings, South Dakota, USA; Vall d'Hebron Research Institute (Ed. Mediterranea)

**Keywords:** *Clostridium difficile*, coculture, colonization resistance, culturomics, fatty acids, metagenomics, microbiome, nutrient competition, pathogen inhibition, phenotypic identification

## Abstract

A major function of the gut microbiota is to provide colonization resistance, wherein pathogens are inhibited or suppressed below infectious levels. However, the fraction of gut microbiota required for colonization resistance remains unclear. We used culturomics to isolate a gut microbiota culture collection comprising 1,590 isolates belonging to 102 species. This culture collection represents 34.57% of the taxonomic diversity and 70% functional capacity, as estimated by metagenomic sequencing of the fecal samples used for culture. Using whole-genome sequencing, we characterized species representatives from this collection and predicted their phenotypic traits, further characterizing isolates by defining nutrient utilization profiles and short-chain fatty acid production. When screened with a coculture assay, 66 species in our culture collection inhibited Clostridioides difficile. Several phenotypes, particularly, growth rate, production of SCFAs, and the utilization of mannitol, sorbitol, or succinate, correlated with C. difficile inhibition. We used a combinatorial community assembly approach to formulate defined bacterial mixes inhibitory to C. difficile. We tested 256 combinations and found that both species composition and blend size were important in inhibition. Our results show that the interaction of bacteria with one another in a mix and with other members of gut commensals must be investigated to design defined bacterial mixes for inhibiting C. difficile *in vivo*.

**IMPORTANCE** Antibiotic treatment causes instability of gut microbiota and the loss of colonization resistance, thus allowing pathogens such as Clostridioides difficile to colonize and causing recurrent infection and mortality. Although fecal microbiome transplantation has been shown to be an effective treatment for C. difficile infection (CDI), a more desirable approach would be the use of a defined mix of inhibitory gut bacteria. The C. difficile-inhibiting species and bacterial combinations identified herein improve the understanding of the ecological interactions controlling colonization resistance against C. difficile and could aid in the design of defined bacteriotherapy as a nonantibiotic alternative against CDI.

## Abstract (precis)

Antibiotic treatment causes instability of gut microbiota and the loss of colonization resistance, thus allowing pathogens such as Clostridioides difficile to colonize and causing recurrent infection and mortality. Although fecal microbiome transplantation has been shown to be an effective treatment for C. difficile infection (CDI), a more desirable approach would be the use of a defined mix of inhibitory gut bacteria. The C. difficile-inhibiting species and bacterial combinations identified herein improve the understanding of the ecological interactions controlling colonization resistance against C. difficile and could aid in the design of defined bacteriotherapy as a nonantibiotic alternative against CDI.

## INTRODUCTION

Normal functioning of the human gut requires a balanced interaction between the mucosal surface, diet, the microbiota, and its metabolic by-products. A major determinant of gut homeostasis is the presence of a healthy, diverse commensal microbiota, which prevents pathogenic bacteria from colonizing the gut or keeps their numbers below pathogenic levels. This function of the gut microbiome is called colonization resistance (1, 2). Perturbations in the gut microbiome, referred to as dysbiosis, can result in the loss of colonization resistance (3). Dysbiosis and loss of gut microbiome colonization resistance caused by antibiotics, for example, can predispose people to enteric infections. Clostridioides difficile infection (CDI) of the gut following antibiotic treatment is a clear demonstration of this phenomenon. Clostridioides difficile is a Gram-positive spore-forming anaerobe that is the leading cause of antibiotic-induced diarrhea in hospitalized patients (4).

Antibiotic treatment of CDI often causes recurrence (5). Infusion of the fecal microbiome from a healthy person into the gut of a patient with CDI can resolve CDI and prevent recurrence (6, 7). This procedure, termed fecal microbiome transplantation (FMT), has become a common treatment for CDI (8). However, concerns have been raised regarding the long-term health consequences of FMT. Recently, weight gain (9) and mortality have been reported as a result of transfer of multidrug-resistant organisms after FMT (10).

The development of defined bacterial mixes that are derived from the healthy microbiota which can resolve CDI may provide an alternative to FMT (11). However, the exact number of species needed in an efficacious defined bacterial mix for CDI remains unknown but has been reported to be in the range of 10 to 33 species when defined bacteriotherapy was tested in a limited number of patients (12, 13). A mix of spore-forming species tested in phase II clinical trials, despite initial success, later resulted in recurrence (14). The use of high-throughput anaerobic gut bacterial culturing coupled with sequencing improves the cultivability of the gut microbiota (15–17), thus facilitating the development of culture collections of gut commensals that can be screened either to identify species conferring colonization resistance or to provide an understanding of the ecological interactions that stabilize or destabilize colonization resistance.

Here, we report the cultivation, using culturomics, of 1,590 gut commensals comprising 102 species from healthy human donors. We phenotyped and sequenced genomes of the representative species in this culture collection. We then screened the strains to identify species inhibiting C. difficile. A combinatorial community assembly approach was used, in which 256 strain combinations were tested to identify the species interactions that improved or diminished C. difficile inhibition. Our results showed that species composition and interactions are both important determinants of the C. difficile inhibition phenotype. Our approach and culture library, in addition to advancing the understanding of bacterial community interactions determining colonization resistance, may prove useful in other studies probing the role of gut microbiota in host health.

## RESULTS

### Single-medium-based culturomics retrieves high species diversity from donor fecal samples.

**FIG 1:**
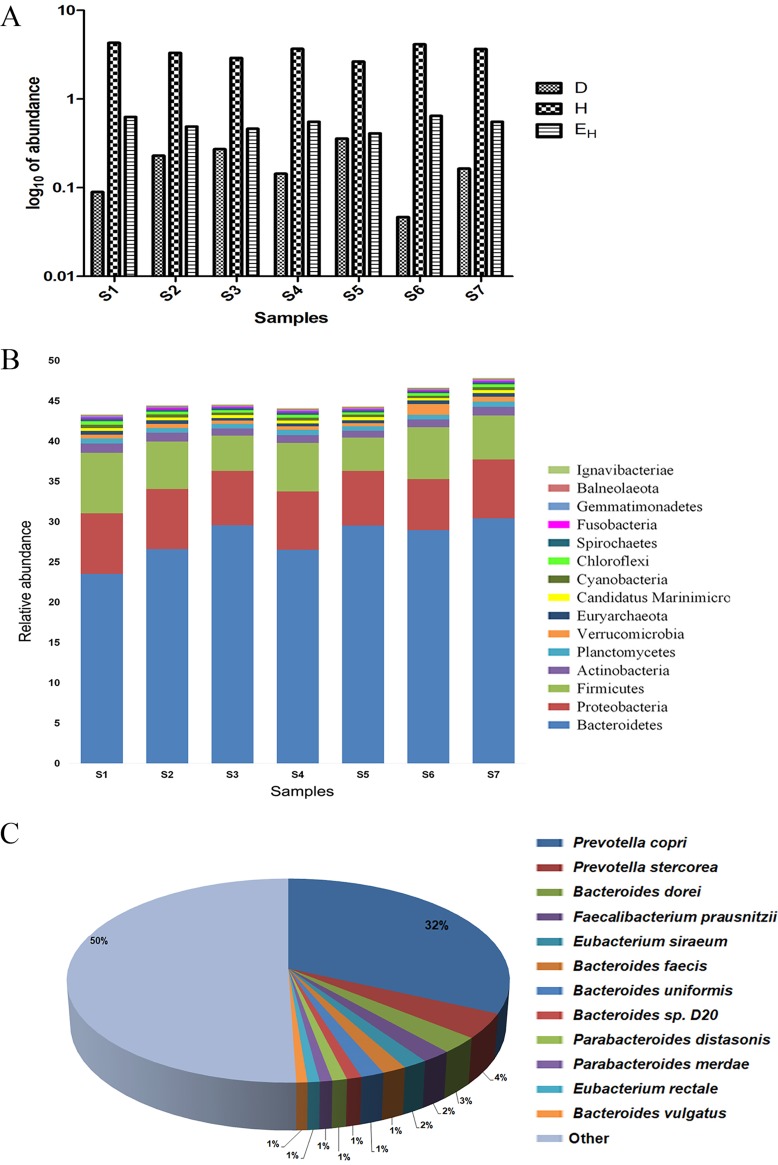
Gut microbiome compositions of donor fecal samples. (A) Variations in the Simpson dominance (D), Shannon diversity (H), and Shannon equitability (E_H_) indices calculated on the basis of the raw reads for taxonomic affiliations for individual fecal samples (S1 to S6) and the pooled fecal sample (S7). (B) Phylum-level distributions of bacteria for individual fecal samples (S1 to S6) and the pooled fecal sample (S7). We calculated relative abundance values compared to the total identifiable reads from any data set. (C) Species-level distribution of the pooled donor fecal sample (S7). We performed taxonomic classification with Kaiju. We placed all species with less than 1% abundance in the “other” category.

In this study, we used metagenome sequencing to characterize the fecal microbiome composition of healthy human donors and used culturomics to develop a strain library to identify C. difficile-inhibiting species. In the first step, donor fecal samples were characterized with shotgun metagenome sequencing. To this end, fecal samples from six donors were sequenced individually and after pooling in equal proportions. We used a high sequencing depth for the metagenome sequencing. Collectively, the data sets from all samples constituted 48.9 Gb of data. The Simpson dominance index (D), Shannon diversity index (H), and Shannon equitability index (E_H_) were calculated for individual samples as well as for the pooled material. This analysis (Hutcheson *t* test, *P* > 0.1) revealed that all donor samples had similar diversity indices, and pooling the samples in equal proportions maintained the overall population structure of the individual samples (Fig. 1A). The donors in this study were recent migrants to the United States from Asia and were expected to have a high proportion of *Prevotella* (*Prevotella* enterotype) in the gut microbiome. In agreement with this expectation, taxonomic diversity of the samples at the phylum level (Fig. 1B) showed the dominance of *Bacteroidetes*, whereas the most abundant genus was *Prevotella* (Fig. 1C).

**FIG 2:**
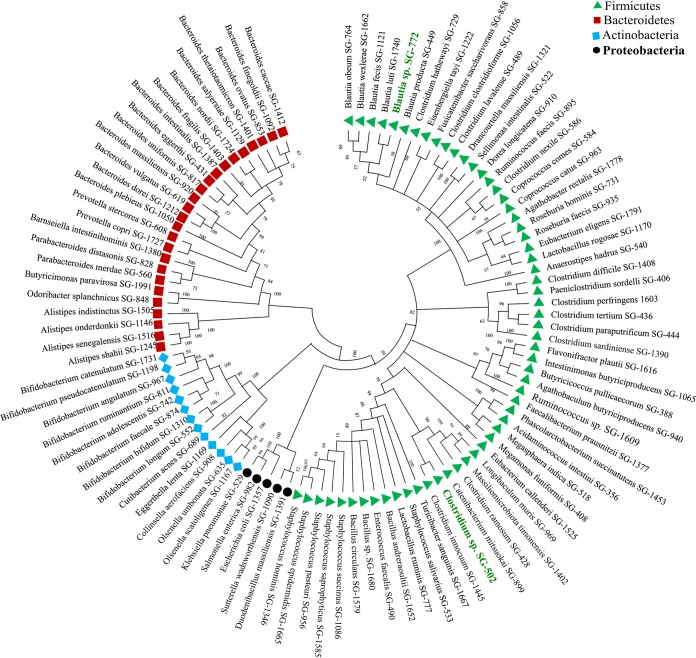
Neighbor-joining tree of the full-length 16S rRNA gene sequences of 102 cultured species isolated from the pooled donor fecal sample. We computed the evolutionary distances with the Jukes-Cantor method; results are presented as the number of base substitutions per site in MEGA6. Symbols and colors represent four different bacterial phyla, as referred to in the legend. Putative novel species (*n* = 2) are indicated with green text.

**FIG 3:**
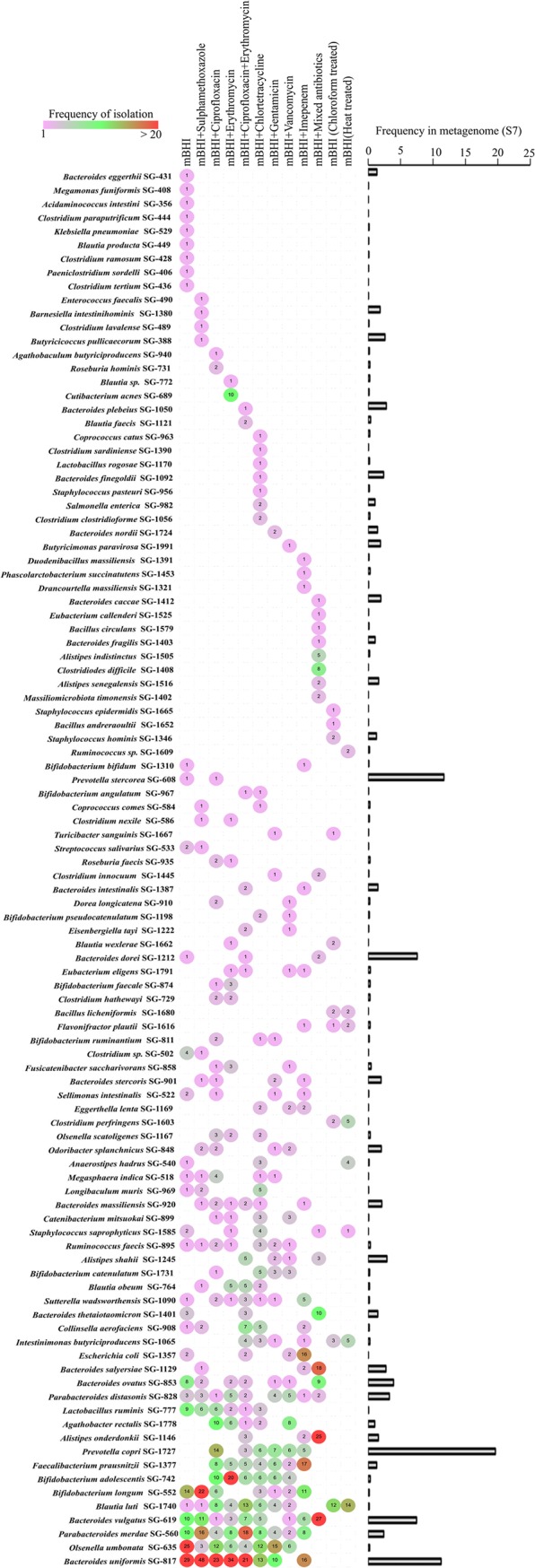
Efficiency of species retrieval under the culture conditions tested. (Left) Frequency of isolation of 102 species recovered. (Right) Prevalence of individual species, as found from read mapping against donor fecal metagenome.

Using culturomics, we developed a strain library from the pooled fecal samples. Previous studies have used various media to isolate gut bacteria (15). For mechanistic studies aimed at understanding the microbial community interaction, strains must be pooled in a single nutrient medium. Although using different medium conditions is useful in retrieving high diversity, strains isolated under various medium conditions may not be able to grow in a single given medium, thus preventing the use of a culture library for a community assembly studied in a universal medium. To avoid this problem, we used a modified brain heart infusion (mBHI) as the base medium for culturing. We isolated several strains from nine species from the mBHI medium. We reasoned that if the species that grew rapidly in the mBHI medium were suppressed, additional species diversity could be isolated using the same medium. We therefore supplemented mBHI with different combinations of antibiotics selected to suppress the formerly dominant strains, and we also used heat shock and chloroform treatment to select for spore-forming species. We used 12 conditions for culturing (see Text S1 in the supplemental material) and selected 1,590 colonies from these conditions. We determined strain species identity with matrix-assisted laser desorption ionization–time of flight (MALDI-TOF) and 16S rRNA sequencing (see Table S1). We thus isolated 93 more species from the same sample, increasing the total diversity in our strain library to 102 species (Fig. 2). In Fig. 3A, we present the frequency of each species isolated under each culture condition. We further examined whether our approach could isolate high- and low-abundance species from the sample. We therefore sequenced the genome of one representative isolate from each species in our library (see Table S2) and mapped the metagenome reads (see Table S3) against the individual species genomes. We mapped 37,669,789 reads obtained from the pooled sample to species whole genomes (Fig. 3B), matching 19,109,642 reads—34.57% of the total metagenomic diversity—to the metagenomic reads. Our single-medium-based culturomics method was able to isolate approximately 34.57% of the pooled culture sample diversity. We mapped approximately 20% of reads against the Prevotella copri SG-1727 genome isolated under six different conditions; this result was unsurprising, given that the fecal donors had the *Prevotella* enterotype. However, we isolated low-abundance species such as Olsenella umbonata, for which only 0.5% of reads were mapped, under eight different conditions (Fig. 3B). We isolated both low- and high-abundance species with our technique and used metagenome binning to estimate the number of species missed by our method. Matching metagenome bins against cultured species genomes, we found that 50 matching culture isolates and 33 bins had no matches, thus indicating that our method did not cultivate those metagenome bins (see Table S4).

10.1128/mSystems.00620-19.1 TEXT S1 Supplemental materials and methods. Download Text S1, DOCX file, 0.05 MB. Copyright © 2020 Ghimire et al. 2020 Ghimire et al. This content is distributed under the terms of the Creative Commons Attribution 4.0 International license.

10.1128/mSystems.00620-19.4 TABLE S1 Summary of 1,590 bacterial isolates identified in the culturomics study. Download Table S1, XLSX file, 0.2 MB. Copyright © 2020 Ghimire et al. 2020 Ghimire et al. This content is distributed under the terms of the Creative Commons Attribution 4.0 International license.

10.1128/mSystems.00620-19.5 TABLE S2 Summary of the genomic features and sequence accession numbers for 102 bacterial species isolated with culturomics from the pooled donor fecal sample. Download Table S2, XLSX file, 0.02 MB. Copyright © 2020 Ghimire et al. 2020 Ghimire et al. This content is distributed under the terms of the Creative Commons Attribution 4.0 International license.

10.1128/mSystems.00620-19.6 TABLE S3 Summary of metagenomic investigation of the six donors (S1 to S6) and pooled donor (S7) fecal sample and their assembly statistics. Download Table S3, XLSX file, 0.01 MB. Copyright © 2020 Ghimire et al. 2020 Ghimire et al. This content is distributed under the terms of the Creative Commons Attribution 4.0 International license.

10.1128/mSystems.00620-19.7 TABLE S4 Comparison of the 102 species isolated in culture and high-quality bins obtained from metagenomes (S1 to S7) from this study, with the most abundant 71 species and 20 species reported by Costea et al. (18) and Forster et al. (16), respectively. The numbers 1 and 0 denote the presence and absence of the bacteria in the studies. Download Table S4, XLSX file, 0.01 MB. Copyright © 2020 Ghimire et al. 2020 Ghimire et al. This content is distributed under the terms of the Creative Commons Attribution 4.0 International license.

### Single-medium-based culturomics retrieves the most frequent gut bacteria in human populations and increases the gene repertoire in the integrated gene catalog of the gut microbiome.

The availability of high-quality metagenome data from many human samples facilitates the identification of frequently present gut bacterial species in healthy populations. A recent study has defined 71 bacterial species present across 2,144 human fecal metagenomes (18). To quantify the number of these species present in our culture library, we matched the whole genomes of 102 bacterial species and 33 metagenome bins against these 71 frequent species (18) and found that our culture library contained 65 of these most frequent bacteria (Table S4). To determine the relationship between the gene repertoire in our culture library and the integrated gene catalog (IGC) of the human microbiome, we compared the two data sets. For the comparison against the IGC, a nonredundant gut microbiome gene set of 9.879 million genes generated from 1,267 human gut metagenomes from Europe, America, and China (19), we generated a nonredundant gene set from our cultured genomes and the sample metagenome used for culturing. We created 984,515 open reading frames (ORFs) from sample metagenomes and 285,672 ORFs from cultured species genomes (see Fig. S1A and Table S3) and mapped them against the IGC. Of 285,672 ORFs obtained from cultured genomes, we matched 208,708 ORFs (73.05%) to the IGC, whereas 572,437 ORFs (58.14%) of 984,515 ORFs obtained from the sample metagenome matched the IGC (Fig. S1B and C). In other words, the IGC lacked 26.94% of the ORFs from the cultured isolates and 41.85% of the ORFs from the sample metagenome. This result demonstrates the potential for expansion of the IGC if more *Prevotella* enterotype donor fecal samples, such as those used in our study, were sequenced. Our results also show that sequencing cultured species genomes identifies genes otherwise missing in metagenome sequencing because of low depth or assembly issues.

10.1128/mSystems.00620-19.2 FIG S1 (A) Numbers of nonredundant ORFs predicted in 102 cultured species and donor fecal metagenomes. We generated the number of nonredundant ORFs at a 95% identity cutoff for donor metagenomes and 102 isolates. (B) Comparison of the nonredundant ORFs generated from 102 cultured species with the existing integrated human gene catalog (IGC). (C) Comparison of the nonredundant ORFs generated from donor metagenomes with the existing IGC. Download FIG S1, TIF file, 3.0 MB. Copyright © 2020 Ghimire et al. 2020 Ghimire et al. This content is distributed under the terms of the Creative Commons Attribution 4.0 International license.

### A large number of species in the culture library inhibit C. difficile *in vitro*.

**FIG 4:**
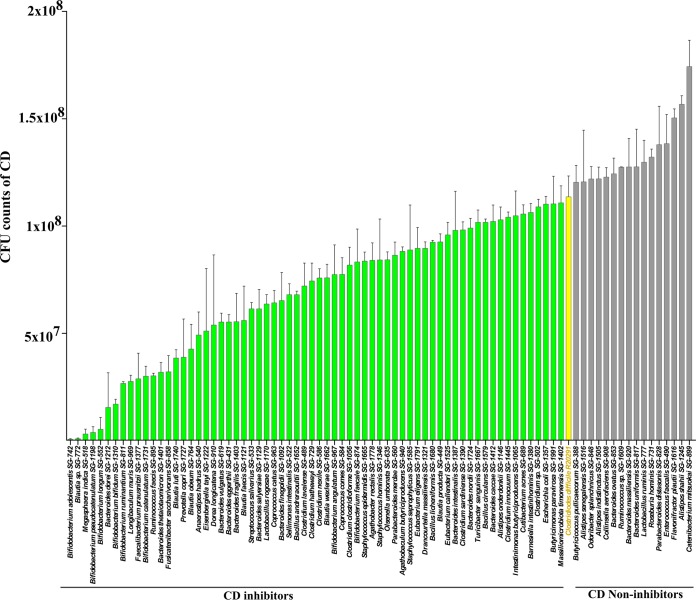
Inhibition of C. difficile by individual species *in vitro*. C. difficile CFU counts were plotted for every individual coculture assay performed for 82 test species in triplicates. Error bars represent standard deviations from the three independent experiments for each inhibition assay for individual species. Green, yellow, and gray bars represent CFU counts of C. difficile for inhibitors, control C. difficile, and noninhibitors, respectively, in coculture.

A healthy microbiota suppresses pathogen growth in the gut. To identify C. difficile-inhibiting species in our culture library, we screened it against C. difficile by using coculture assays. Since slow-growing strains would be outcompeted by C. difficile, we used 82 moderately or fast-growing species in the coculture assay. When tested, 66 species inhibited C. difficile to various degrees (Fig. 4). In this screen, Bifidobacterium adolescentis strain SG-742 was the most efficient inhibitor. Furthermore, all bifidobacteria inhibited C. difficile, thus indicating their importance in colonization resistance to this pathogen. The *Lachnospiraceae* family were major inhibitors. Unexpectedly, 16 species in our coculture assay increased the growth of C. difficile (Fig. 4), a finding with potential clinical importance, because high abundance of these species may confer a greater risk of CDI.

### Most inhibitors are acetate or butyrate producers.

**FIG 5:**
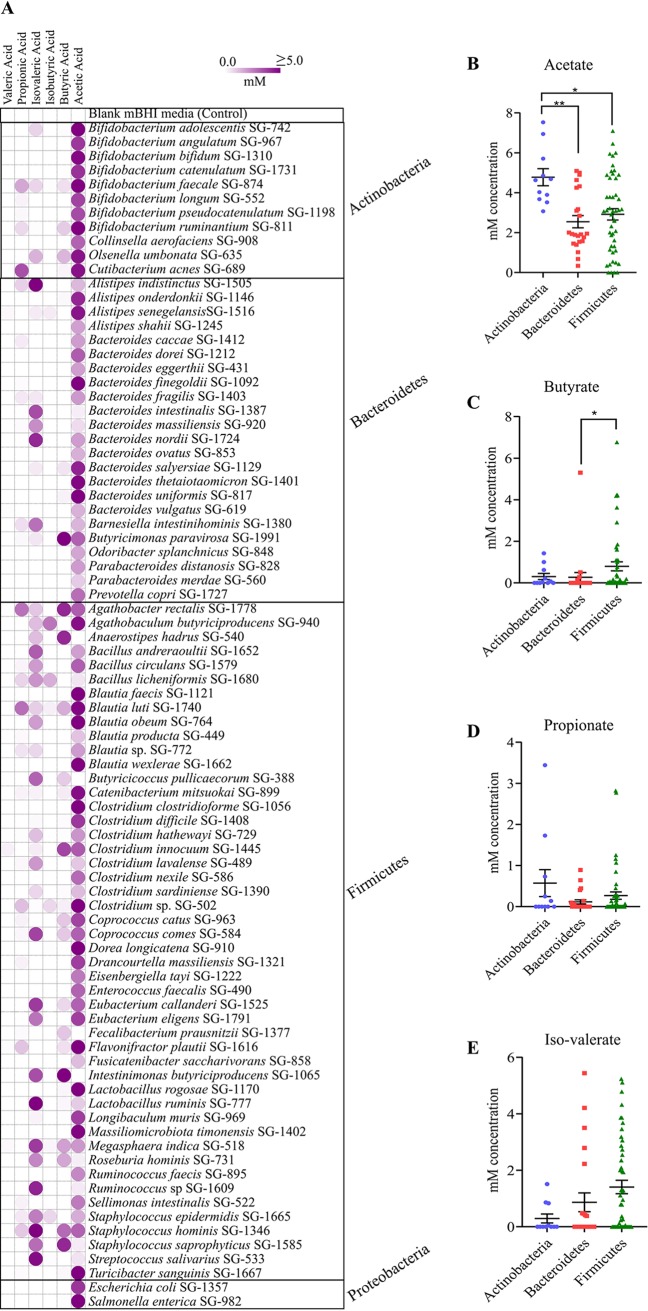
Short-chain fatty acids (SCFAs) produced by cultured species. (A) SCFA production by 82 species used in inhibition assays against C. difficile. We measured acetate, propionate, isobutyrate, butyrate, isovalerate, and valerate with gas chromatography; results are expressed in millimolar concentrations. The figure represents mean SCFA measurements from duplicate samples. Color scale bars: white, no SCFA production; dark, ≥5 mM SCFA production. Acetate (B), butyrate (C), propionate (D), and isovalerate (E) production in three major phyla from 80 bacteria, oriented according to phyla (Kruskal-Wallis tests, *P* < 0.05). SCFA levels for two members of *Proteobacteria* phyla are not shown in B, C, D, and E.

Gut bacteria metabolize diverse substrates and produce short-chain fatty acids (SCFAs) in the gut (20, 21). SCFAs, particularly butyrate, act as gut epithelial immune modulators, energy sources for host intestinal cells, and pathogen inhibitors (22, 23). To determine the relationship between the SCFAs produced and C. difficile inhibition, we estimated the SCFAs produced by all species used in the coculture assays (Fig. 5). Our results showed that the strains produced mainly acetate (Fig. 5A; see also Table S5). Comparing all strains at the phylum level by using the nonparametric Kruskal-Wallis test, we found that *Actinobacteria* and *Bacteroidetes* yielded the most acetate (Fig. 5B). *Firmicutes* produced significantly more butyrate than *Bacteroidetes* (Fig. 5C). The production of other SCFAs did not differ significantly (Fig. 5D and E). Most high acetate or butyrate producers were C. difficile inhibitors.

10.1128/mSystems.00620-19.8 TABLE S5 Estimation of SCFAs produced by 82 species of commensals for which we performed inhibition assays against C. difficile R20291. Download Table S5, XLSX file, 0.01 MB. Copyright © 2020 Ghimire et al. 2020 Ghimire et al. This content is distributed under the terms of the Creative Commons Attribution 4.0 International license.

### Relationships among nutrient utilization, C. difficile inhibition, and the prevalence of other species in patient populations.

**FIG 6:**
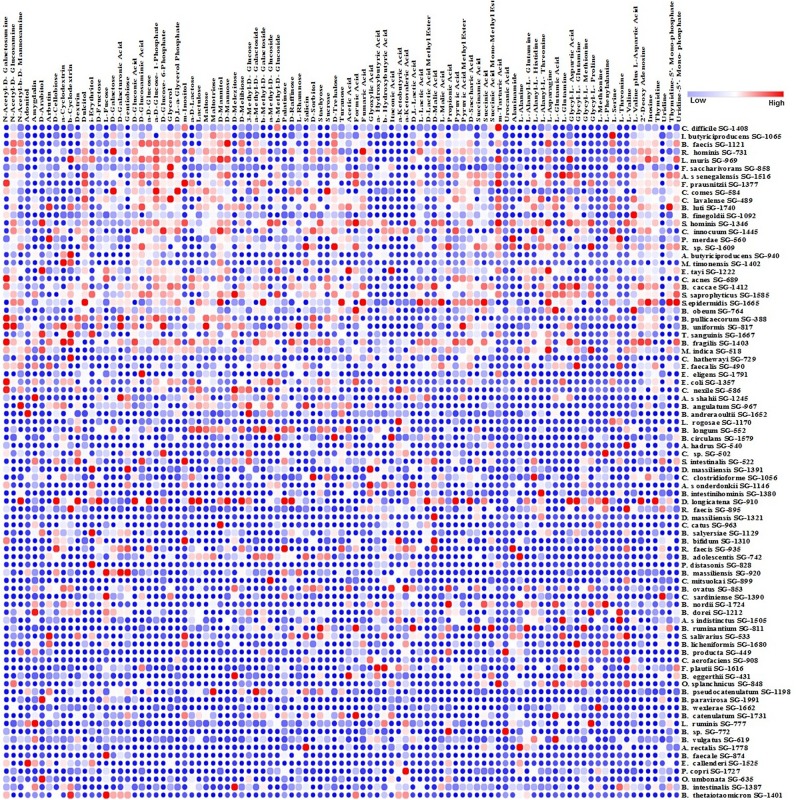
Utilization of 95 nutrient sources by the cultured species. The heat map represents two independent substrate utilization tests normalized against the control. Columns and rows represent nutrients and strains, respectively. We considered growth of ≥20% in any substrate compared with the control as positive. Blue, white, and red represent low, moderate, and high utilization of the carbon source, respectively. The top row shows nutrient utilization of C. difficile. All other strains are arranged in descending order of nutrient utilization similarity to that of C. difficile. We used nearest neighbor clustering based on the Pearson correlation to identify the nutrient utilization similarity of other strains compared with C. difficile.

**FIG 7:**
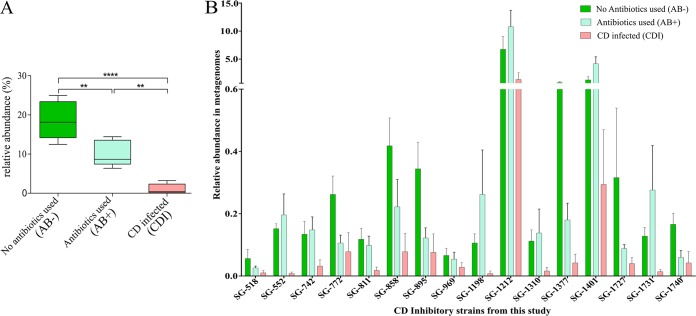
Frequency of the top 25% C. difficile inhibitors obtained in this study in the gut microbiome of participants with and without CDI. AB+ and AB− represent samples from patients without CDI who were treated with antibiotics and patients without CDI who received antibiotic treatment, respectively. (A) The combined abundance of the top 25% C. difficile inhibitors (*n* = 16) from this study in CDI, AB+, and AB− metagenomes. (B) Individual abundances of the same 16 strains in CDI, AB+, and AB− metagenomes. We obtained public metagenomes for CDI, AB+, and AB− from Milani et al. (29).

Commensal species suppress pathogens in the gut chiefly by competing for nutrients (24–26). The Biolog AN MicroPlate test panel provides a standardized method to identify the utilization of 95 nutrient sources by anaerobes. Investigating the relationship between nutrient utilization and pathogen inhibition, we determined the nutrient utilization of all species in our library. C. difficile is known to exploit mannitol, sorbitol, or succinate to invade and produce infection in the human gut (27, 28). In agreement with this observation, we found 27 C. difficile inhibitors that used all three nutrients as carbon sources (Fig. 6). We hypothesized that if the C. difficile inhibitors we identified were active in C. difficile suppression *in vivo*, their abundance would decrease in the gut during antibiotic treatment and CDI. Hence, we determined the abundance of the top 16 (top 25%) C. difficile inhibitors in our library from a study of gut microbiomes from healthy individuals and patients with CDI (29). We determined the frequency of the top 16 C. difficile-inhibiting isolates in the following groups of human gut metagenomes: (a) patients with CDI, (b) patients who were antibiotic exposed but did not have CDI, and (c) people with no antibiotic exposure and no CDI (healthy). In agreement with our hypothesis, the top 16 C. difficile inhibitors in our screen constituted approximately 20% of the total abundance in the heathy microbiome but had low abundance in both the antibiotic-treated and CDI patient gut samples (Fig. 7A). Interestingly, Prevotella copri (SG-1727)—among the most abundant species in the donor samples—was one of the species depleted during antibiotic treatment and CDI (Fig. 7B).

### Analysis of genotype-phenotype relationships.

**FIG 8:**
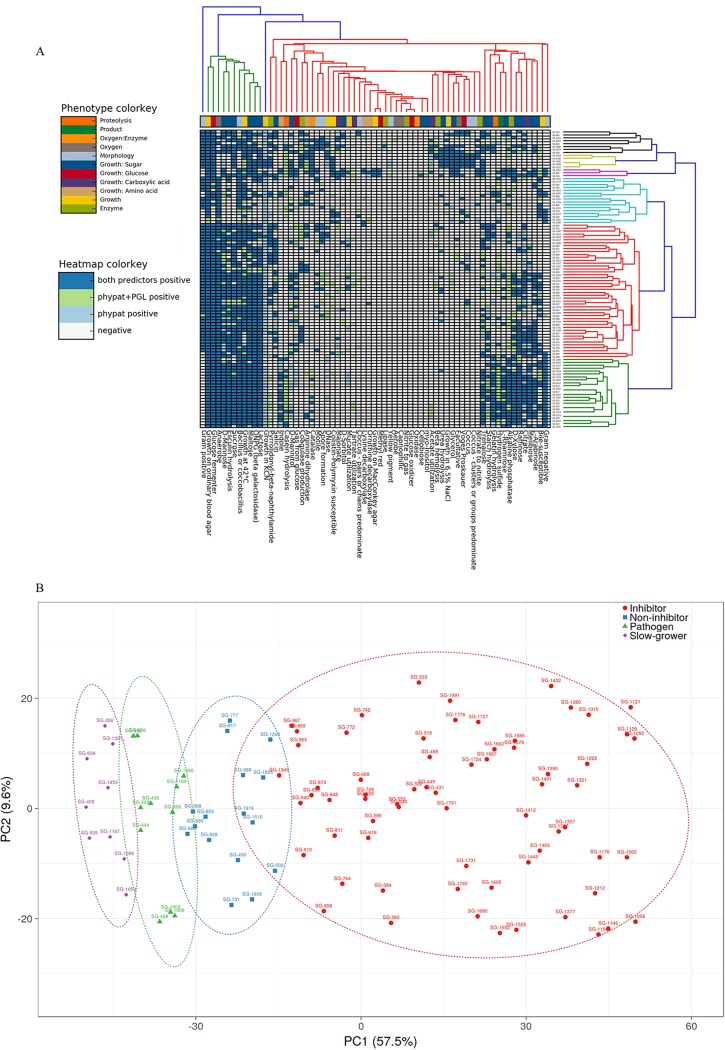
Prediction of phenotypes from the genomes of the 102 species in the culture library. (A) Clustering of 102 species on the basis of 67 traits predicted with the Traitar package. Each column represents one of 67 traits, whereas rows represent 102 species from this study. The color scheme of the columns further depicts 11 phenotypic properties from proteolysis to enzyme production. (B) PCA using the combined predicted traits from Pfam annotation. The *x* and *y* axes show principal components 1 and 2, explaining 57.5% and 9.6% of the total variance, respectively. Prediction ellipses are based on 0.95 confidence. The color scheme in the legend represents four different categories of isolates.

To identify other phenotypes not covered in our phenotype assays and to link phenotype with genotype, we used Traitar (30) to predict 67 phenotypes from the genomes of all species in our library. The substrate utilization phenotypes based on the Traitar prediction mostly matched the Biolog phenotypes (Fig. 8A; see also Table S6). The first two clusters (green and red, lower Fig. 8A) primarily comprised the pathogen inhibitors tested herein. The defining traits in these clusters were associated with sugar hydrolysis, mostly matching the Biolog phenotype data. The other two clusters (sky blue and mixed) comprised mostly pathogenic species and slow growers. Notable traits for pathogen clusters were catalase activity, beta hemolysis activity, growth in glycerol, and high osmotolerance (Fig. 8A). To further differentiate these traits, we performed principal-component analysis (PCA) on the Traitar data. The PCA plot (Fig. 8B) revealed four distinct clusters (C. difficile inhibitors, noninhibitors, pathogens, and slow-growing strains) that explained 67.1% total variance in the first two principal components. Slow-growing strains clustered furthest from C. difficile-inhibitors, a finding that might be expected for any colonization-resistant bacteria, because a high growth rate would be more likely to outcompete the pathogen.

10.1128/mSystems.00620-19.9 TABLE S6 Prediction of species phenotypes from the genomes with the Traitar package. Each column represents one of 67 traits, whereas rows represent 102 isolates from this study. 0, no trait predicted; 1, trait predicted by the Phypat algorithm alone; 2, trait predicted by the PGL algorithm alone; 3, trait predicted by both the Phypat and PGL algorithms. Download Table S6, XLS file, 0.1 MB. Copyright © 2020 Ghimire et al. 2020 Ghimire et al. This content is distributed under the terms of the Creative Commons Attribution 4.0 International license.

To understand the genomic basis of colonization-resistant and pathogen-inhibiting strain genomes, we used KEGG modules, which are characteristic gene sets that can be linked to specific metabolic capacities or other phenotypic features of a genome. We identified 515 modules in the sample metagenome and 476 modules in the strain genomes. We found that 432 modules were common between our sample metagenome and our strain genomes, thus demonstrating our ability to retrieve 77.28% of the metabolic functional capacity of the fecal microbiome with our culture method. The 82 modules unrecovered in our isolated species comprised pathways associated with environmental information processing, several components of cell signaling, DNA replication and repair pathways, lipid metabolism, RNA and protein processing, and the ubiquitin system (see Fig. S2). We then sought to identify differences in KEGG modules associated with pathogen-inhibiting and pathogen-noninhibiting strain genomes and found that 26 modules that were present in C. difficile inhibitors were absent in non-C. difficile inhibitors. Some important modules absent in noninhibiting species genomes were M00698 (multidrug resistance efflux pump BpeEF-OprC), M00332 (type III secretion system), M00438 (nitrate/nitrite transport system), and M00551 (benzoate degradation). Further work is necessary to understand how these functional modules relate to colonization resistance.

10.1128/mSystems.00620-19.3 FIG S2 Hierarchical clustering of KEGG modules from the fecal sample metagenome, all 102 species (All_isolates) and subsets found to inhibit C. difficile R20291 (CD_inhibitors). We annotated predicted ORFs from the pooled donor fecal metagenome and consortium of cultures for KO modules by searching against the KEGG database with GhostKOALA. Completeness of the KEGG modules is indicated by the color gradient (from 0 indicating a complete module to 4 indicating absence of a whole module). Download FIG S2, JPG file, 0.8 MB. Copyright © 2020 Ghimire et al. 2020 Ghimire et al. This content is distributed under the terms of the Creative Commons Attribution 4.0 International license.

### Design of a defined mix of C. difficile inhibitors.

**FIG 9:**
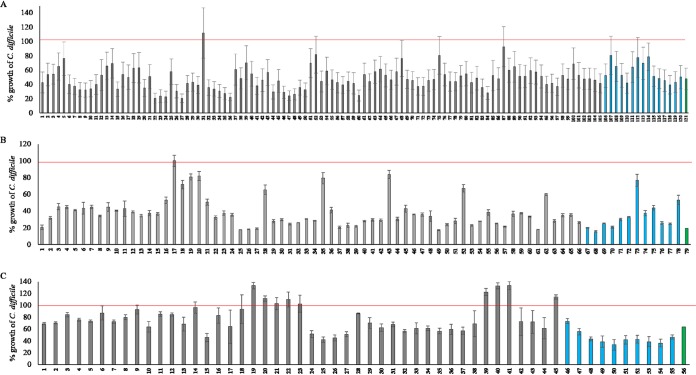
C. difficile inhibition efficiency of the consortia. (A) Combinations starting with a 15-species pool. (B) Combinations starting with 12-species pool. (C) Combinations starting with a 10-species pool. Green, blue, and gray bars represent the parent blend, the blend with one bacterial species removed at a time, and the blend with two bacterial species removed at a time, respectively. The red lines represent C. difficile control growth. We normalized the growth of C. difficile in each consortium to the control C. difficile growth to obtain the relative C. difficile growth (represented as percentage C. difficile growth) under each consortium condition. We performed each experiment in triplicates, and error bars represent the standard errors of the means.

While single strain versus pathogen coculture assays are informative in identifying pathogen-inhibiting strains, the inhibition patterns are likely to change when inhibiting species interact as a community. These communities may express emergent properties that are difficult to predict from the individual members (30). After defining the isolate phenotypes, we used a combinatorial assembly of bacteria from our culture collection to design a tractable mix of C. difficile*-*inhibiting isolates. In the first set of experiments, we mixed 15 inhibiting species in equal proportions and tested them against C. difficile using the coculture assay used for individual strains. Table 1 describes the overall properties of these isolates, which were selected on the basis of the criterion that the medium pH did not drop below 5.6 after 24 h of growth, and the representation of overall taxonomic diversity at the family level. To investigate how changes in the mix composition might affect the inhibition capacity, we removed one or two species at a time from the mix of 15 species to create additional mixes, thus testing 121 mixes listed in Table S7 against C. difficile in coculture assay format. As shown in Fig. 9, the removal of strains from the 15-species mix had both positive and negative effects. When we removed species, several mixes were less effective than the 15-species mix, and the removal of two species increased the inhibition efficiency in a species-dependent manner. Of 121 mixes tested, mix number 22, comprising the species listed in Table S7, most effectively inhibited C. difficile growth (by 79.41%). This finding clearly demonstrates the dependence of inhibition efficiency on the species composition of the defined mix used in the coculture assay. Seeking the minimum number of species necessary for an effective C. difficile*-*inhibiting mix, we performed another set of experiments in which either one or two species at a time were removed from the parent mix of 12 species. In this round, we tested 79 bacterial mixes comprising species listed in Table S7 in the coculture assays. As shown in Fig. 9, removal of species primarily decreased the inhibition efficiency relative to that of the parent blend. Again, the efficiency was dependent on the species composition of the mix. We performed a third set of experiments to determine mixes comprising fewer than ten species affecting C. difficile inhibition efficiency. Removal of species from the ten-species parent set diminished inhibition efficiency overall; however, some mixes increased the growth of C. difficile. Because all the strains that we used individually inhibited C. difficile, the enhancement of C. difficile growth by these set mixes clearly demonstrates that an individual strain phenotype can be overridden by species community interactions. In this case, a set of C. difficile-inhibitory species, when mixed in a particular combination, increased C. difficile growth. Overall, our results demonstrate that new phenotypes masking the individual strain phenotype can emerge when microbial consortia are formed, and this emergent property must be considered in designing defined C. difficile-inhibiting bacterial mixes.

**TABLE 1:** Strains used in the combinatorial mix assembly

Strain	Family	C. difficile inhibition^*a*^	pH after growth in mBHI	No. of carbon sources used
Bifidobacterium bifidum SG-1310	*Bifidobacteriaceae*	***	6.333	30
Bacteroides eggerthii SG-431	*Bacteroidaceae*	**	6.100	35
Bacteroides finegoldii SG-1092	*Bacteroidaceae*	**	5.713	62
Bacteroides vulgatus SG-619	*Bacteroidaceae*	**	5.917	70
Parabacteroides merdae SG-560	*Tannerellaceae*	**	5.913	74
Prevotella copri SG-1727	*Prevotellaceae*	***	6.013	39
Lactobacillus rogosae SG-1170	*Lactobacillaceae*	**	6.003	22
Clostridium nexile SG-586	*Lachnospiraceae*	**	6.307	32
Eubacterium eligens SG-1791	*Eubacteriaceae*	**	6.237	26
Blautia wexlerae SG-1662	*Lachnospiraceae*	**	5.733	34
Sellimonas intestinalis SG-522	*Lachnospiraceae*	**	6.103	49
Drancourtella massiliensis SG-1321	*Ruminococcaceae*	**	5.770	5
Megasphaera indica SG-518	*Veillonellaceae*	***	5.640	75
Bacillus licheniformis SG-1680	*Bacillaceae*	**	5.927	35
*Clostridium* sp. SG502	*Erysipelotrichaceae*	*	5.880	30

a ***, high inhibition; **, moderate inhibition; *, low inhibition.

10.1128/mSystems.00620-19.10 TABLE S7 Species composition of bacterial consortia tested against C. difficile. For each experiment, we generated a parent blend, a blend with one bacterial strain removed at a time, and a blend with two bacterial strains removed at a time. The blend numbers correspond to those in Fig. 9. Download Table S7, XLSX file, 0.03 MB. Copyright © 2020 Ghimire et al. 2020 Ghimire et al. This content is distributed under the terms of the Creative Commons Attribution 4.0 International license.

## DISCUSSION

We developed a gut commensal culture collection from healthy human donors and identified C. difficile colonization-resistant strains. Because the human gut microbiome composition varies across populations, donor selection for culture is an important consideration. Depending on the proportion of *Bacteroides* and *Prevotella*, the human gut microbiome has been classified into enterotypes (31). The Asian and African populations—two-thirds of the human population—fall into the *Prevotella* enterotype (32). Information about the colonization-conferring species in *Prevotella*-dominant gut microbiomes is limited; we therefore chose recent Asian immigrants in the United States as the fecal donors in this study. For culture, we used a pooled sample from six donors before culturing, which could have had positive and negative consequences. Pooling can save substantial time and resources. For instance, after pooling, we analyzed 1,590 colonies; had analyses been performed individually, the number of analyzed colonies would have been 9,540. Pooling, however, may distort the microbiome composition of individual samples, thereby creating artificial population assemblages (33). For gut microbiome ecology studies, a gut commensal culture collection isolated from fecal donors of similar gut microbiome compositions could be more useful than one from many different people. The publicly available gut microbiota culture collection from the human microbiome project was isolated from 265 people (34), and other similar culture collections have been isolated from at least 100 donors (15, 17). Although such collections are useful as reference strains, because the donors may have different microbiome compositions, the strains isolated may not form stable ecologies if mixed. In contrast, our collection, from a limited number of donors with similar microbiome compositions, may have formed a stable ecology when mixed and consequently might be more useful in studies seeking to understand the underlying interactions determining colonization resistance and other traits.

Two general approaches have been used for developing gut microbiota culture collections: first, culturing samples in several—often as many as 64—different nutrient medium conditions, to isolate diversity (15, 35, 36) and second, the use of a single medium, requiring less time and fewer resources, but retrieving fewer species (37). The use of different media is more efficient in isolating slow-growing species that are often biased against in the medium, such as BHI. In understanding the types of bacterial communities responsible for suppressing pathogen growth in the gut, assembling simple to complex bacterial communities from cultured strain libraries and testing the consortia against pathogens are commonly performed. Although the use of different nutrient media is highly efficient in isolating the maximum number of species from gut samples, strains so isolated may not grow in a common nutrient medium, thus diminishing the utility of the strains in community assembly studies. We therefore used a single-medium-based approach with mBHI for culturing the fecal bacteria. As shown in Fig. 2 and 3, we isolated 102 species (representing approximately 34.57% species diversity, as determined by sequencing the donor fecal sample) by adjusting the mBHI medium (see Fig. S1 in the supplemental material); this process was comparable to other single-medium-based approaches (37–39). Furthermore, the 34.57% diversity isolated represents >70% functional capacity of the donor gut microbiome (Table S3; Fig. S2). According to the insurance hypothesis of microbiota function, more than one species performing the same function is recruited in an ecosystem to allow for functional redundancy (40, 41), thus possibly explaining the recovery of 70% function from 34.57% species diversity in our library.

Many strains in our culture collection shown in Fig. 4 inhibited C. difficile at various levels. Several phenotypes, particularly, growth rate, production of SCFAs, and the utilization of mannitol, sorbitol, or succinate, correlated with the C. difficile inhibitor phenotype, in agreement with findings from previous reports indicating that restoration of depleted SCFAs in the gut resolved CDI (42, 43) and that competition between C. difficile and commensals for nutrients, and increased availability of mannitol, sorbitol, or succinate, allows C. difficile to invade the gut (27, 28). The top inhibiting species in our collection were also depleted in the gut in patients with CDI, thus indicating their role in providing colonization resistance against C. difficile (Fig. 7). The formation of many different defined bacterial mixes comprising these inhibitory strains may improve the inhibition capacity of the individual strains. Overall, we tested 256 defined mixes using the combinatorial community assembly approach. The combinatorial community assembly method (Fig. 9) showed two important parameters defining the efficacy of the defined mixes: the number and type of species in the mix. Decreasing the number of species in the mix from 15 to 12 did not diminish the overall inhibition capacity but limiting the number to ten species did. Furthermore, many of those mixes increased rather than inhibited C. difficile growth. Clearly, adding too many species in a mix does not improve inhibition. The threshold of peak efficacy was 12 species under the conditions we tested. Our results also underline how undesirable traits can emerge when species are pooled in a suboptimal ecology; strains in combination can produce new phenotypes not observed individually. Previous work to identify a defined mix of C. difficile-inhibiting bacteria has also identified various mix numbers. For instance, more than 20 years ago, Tvede and Rask-Madsen showed that infusion of a mix of 10 bacteria into a patient’s colon can resolve CDI (12). Another study in a small patient population has found that treatment with a mix of 33 bacteria can alleviate CDI. In a mouse model, Clostridium scindens has been found to be a more efficient C. difficile inhibitor when mixed with a defined pool of other commensal bacteria (44). Likewise, a mix of six phylogenetically diverse bacteria has been found to alleviate CDI in a mouse model (45). The complexity of the gut microbiota and its variations across populations make the design of defined bacterial C. difficile-inhibiting mix not simply a matter of mixing large numbers of diverse species. Because our results show that the C. difficile-inhibiting phenotype changes substantially depending on the microbial interaction, designing a defined bacterial mix requires a deeper understanding of how inhibiting species interact with themselves and the members of the total commensal community.

### Conclusion.

Overall, we demonstrated that a high percentage of a fast-growing cultivable fraction of gut microbiota from healthy human donors are C. difficile inhibitors *in vitro*. Defined bacterial mixes can enhance the inhibition capacity of individual strains. However, depending on the ecology of the mix, new phenotypes can emerge. For instance, a mix of bacteria can increase rather than inhibit the growth of C. difficile. In designing defined C. difficile-inhibiting bacterial mixes *in vivo*, the interaction of bacteria with each other in a mix and with other members of gut commensals requires further investigation. The approach of combinatorial testing of strains with well-defined phenotypes used in the present study represents a step in that direction.

## MATERIALS AND METHODS

### Fecal sample collection, culture conditions, isolate identification, and genome sequencing.

We collected fecal samples with the approval of the Institutional Review Board of South Dakota State University. All procedures were performed according to Institutional Review Board guidelines. We collected fresh fecal samples from six healthy adult donors from Brookings, SD, USA, who had not taken antibiotics during the previous year. Subsequently, we transferred fecal samples to an anaerobic chamber within 10 min of voiding, diluted them 10-fold with phosphate-buffered saline, and mixed individual fecal samples in equal ratios to generate the pooled sample for culturing. For culture, we used mBHI broth (ingredients listed in Text S1 in the supplemental material). We used chloroform and heat treatment to isolate spore-forming species, and we plated the 10^4^ dilution of the pooled sample on each of the medium conditions described (Text S1) under strictly anaerobic conditions. We selected 1,590 isolates from all culture conditions and identified them with MALDI-TOF (MALDI Biotyper; Bruker Inc.) against reference spectra (Table S1). We identified isolates that were unidentified at this step through 16S rRNA gene sequencing, for which we prepared total genomic DNA with an OMEGA E.Z.N.A. genomic DNA isolation kit (Omega Bio-Tek, GA) according to the manufacturer’s protocol. We amplified full-length bacterial 16s rRNA genes with universal forward (27F) and reverse (1492R) primers under standard PCR conditions. We sequenced the amplified DNA with the Sanger dideoxy method. We trimmed raw sequences generated for low-quality regions from either end and constructed consensus sequences from multiple primers in Geneious (46) with default parameters. On the basis of full-length 16S rRNA gene sequence similarities, we determined the phylogenetic relationships among the isolates. For bacteria initially identified with MALDI-TOF, we extracted full-length 16s RNA gene sequences from the genome with Barrnap (https://github.com/tseemann/barrnap) for phylogenetic tree creation. The sequences were aligned with MUSCLE (47), and evolutionary distances were computed with the Jukes-Cantor method. Finally, we created a neighbor-joining tree with a bootstrap of 1,000 replicates with MEGA6 (48).

To further characterize the strain library, we selected representative isolates from each species for whole-genome sequencing, extracting genomic DNA from overnight cultures of the isolates by using an OMEGA E.Z.N.A. genomic DNA isolation kit (Omega Bio-Tek, GA) according to the manufacturer’s protocol. We prepared sequencing libraries with a Nextera XT kit and sequenced them with Illumina 2 × 300 paired-end sequencing chemistry on the MiSeq platform (Text S1). We first filtered the raw reads for quality and sequencing adaptors with PRINSEQ (49) and then assembled them *de novo* with Unicycler (50), performing quality checking of the assembly results with QUAST (51) and Bandage (52), all in default mode. We performed gene calling with Prokka (53) with a minimum ORF length of 100 bp.

### Characterization of donor fecal samples using shotgun metagenome sequencing.

We extracted total community DNA from 0.25 g of each donor fecal sample with a MO BIO PowerSoil DNA isolation kit according to the manufacturer’s instructions. To enrich for microbial DNA, using a previously published protocol, we depleted host DNA in the isolated total DNA before sequencing (54). We prepared the sequencing library from 0.3 ng of the enriched DNA with a Nextera XT library preparation kit (Illumina, San Diego, CA), performing sequencing according to the protocols used for bacterial genomes described above. After quality correction, we removed human host reads with Bowtie2 v.1.1.2 (55) and performed taxonomy assignment in Kaiju (56) in greedy mode. We searched reads against the proGenomes (57) reference database of protein sequences containing a nonredundant set from more than 25,000 genomes from every species cluster recovered by specI (58), using the NCBI nonredundant database for comparative analysis. Thereafter, we calculated the Simpson dominance index (D), Shannon diversity index (H), and Shannon equitability index (E_H_), using an assembly-based approach to characterize the donor fecal metagenomes. To do so, we assembled reads *de novo* by using default metaSPAdes (SPAdes 3.12.0) (59), specifically designed for assembly of complex metagenomic communities. For initial assembly, we error corrected reads by using spades-hammer with default parameters and then checked the assembly results with default MetaQUAST v 5.0 (60). We removed contigs of less than 500 bp from the resultant data sets and performed ORF predictions on the filtered contigs with MetaGeneMark (61) with a minimum length cutoff of 100 bp.

### Computation of gene repertoire and the functional analysis of isolate genomes and donor fecal metagenomes.

To determine the gene repertoire in the isolate genomes and donor fecal metagenomes, we constructed a nonredundant gene catalog from our total data by using cd-hit (62, 63) for comparison against the previously published integrated catalog of reference genes in the human gut microbiome (19). After gene calling, we clustered the concatenated data sets from the culture library by using cd-hit at >95% identity with 90% overlap. We checked these data sets with BLAT to avoid overrepresentation in the gene catalog. We then compared our gene data set against the previously mentioned integrated catalog (19). For functional mapping, we mapped amino acid sequences from all the individual data sets and clustered nonredundant sets against the EggNOG database v3.0 (64).

For gaining a better insight into the putative and hypothetical population genomes present within the donor fecal samples but not isolated using the culturomics approach, population genome bin creation is considered superior to taxonomy assignment of the raw reads. We therefore constructed population genome bins from the metagenomes by using MaxBin2 with acceptance criteria of 90% completeness and <5% redundancy, mapping back raw reads on the assembled contigs by using Bowtie2 (65) for coverage information. We further analyzed all high-confidence bins with specI (58) for species cluster determination. To determine the abundance of isolated species from the pooled donor fecal samples, we measured the coverage by read mapping with Bowtie2 (65) at the 95% identity level. For the functional analysis, we performed KEGG annotation for the ORFs obtained from the pooled donor fecal metagenome and the isolate genomes. We searched data from all comparisons for KO modules with GhostKOALA (66) and performed hierarchical clustering of the data sets to generate heat maps with R (http://www.R-project.org/). We used Traitar (67) with default parameters to predict 67 phenotypes from the whole genomes of all species in the culture collection.

### Phenotypic characterization of the isolated strains.

To correlate the genomic features with phenotypes, we further characterized the strains for which genomes were sequenced by determining the following phenotypic properties.

**(i) Carbon source utilization.** We determined the ability to use 95 carbon sources with Biolog AN MicroPlate technology. Briefly, we grew strains on mBHI plates anaerobically for 24 to 48 h at 37°C. We used a sterile cotton swab to scrape cells from the plates and suspended them in AN inoculating fluid (optical density at 650 nm [OD_650_] < 0.02), using 100 μl of this suspension to inoculate an AN MicroPlate in duplicates, and incubated them at 37°C anaerobically. We took OD_650_ readings at 0 h and 48 h postinoculation and normalized the results for growth against water and 0-h OD_650_ values.

**(ii) Production of SCFA.** To analyze the SCFAs produced by isolates, we grew strains in mBHI for 24 h under anaerobic conditions, added 800 μl of the bacterial culture to 160 μl of freshly prepared 25% (wt/vol) *m*-phosphoric acid, and froze the samples at −80°C. We thawed the samples and centrifuged them (>20,000 × *g*) for 30 min. We used 600 μl supernatant for injection into the TRACE1310 GC system (Thermo Scientific, USA) for SCFA analysis.

**(iii) Identification of C. difficile-inhibiting strains.** We used a coculture assay in which the pathogen and test strains were cultured together at a ratio of 1:9 to identify C. difficile*-*inhibiting strains in our library, using only those strains reaching an OD_600_ of 1.5 after 24 h of growth in mBHI; 82 species met this criterion. We used C. difficile strain R20291 as the reference strain in the first assay. Briefly, we grew all test strains and C. difficile R20291 in mBHI medium anaerobically at 37°C and adjusted the OD_600_ to 0.5. The pathogen and the test samples were mixed at a ratio of 9:1 and incubated for 18 h anaerobically at 37°C. We then plated 10^−5^ and 10^−6^ dilutions onto C. difficile selective agar, using a pure culture of C. difficile R20291 as a positive control. We compared CFU enumerated from coculture plates against that from the C. difficile R20291 control. In identifying C. difficile inhibitors in healthy and patients with CDI, we calculated the frequencies of the 16 top C. difficile inhibitors in our collection by metagenome read mapping from a previously published data set (29).

### Data availability.

All metagenomic data sets from this project have been deposited in the NCBI SRA database under BioProject PRJNA494584. Raw whole-genome sequence data for all the isolates have been deposited under BioProject PRJNA494608.
