# Supplementary material for: Identification of Clostridioides difficile-Inhibiting Gut Commensals Using Culturomics, Phenotyping, and Combinatorial Community Assembly
Source: mSystems. 2020 Feb 4;5(1):e00620-19. doi: 10.1128/mSystems.00620-19 (PMC7002114; doi:10.1128/mSystems.00620-19)
Supplement: TEXT S1 [file mSystems.00620-19-s0001.docx]

**Supplementary Text 1**

**Supplementary methods**

*Bacterial culture Medium:* To prepare modified Brain Heart Infusion Medium (mBHI), the following were added to standard BHI base ingredients; yeast extract (5.0 g/L), L-cysteine (0.3 g/L), 1 ml/l Resazurine (0.25 mg/ml) and 1 ml/L Menadione (5.8 mM). Prepared media was transferred to 500 ml Hungate capped bottles and purged with nitrogen at high temperature until the media became completely anaerobic which was indicated by the absence of pink color by indicator resazurine. that the medium was autoclaved at 121^0^C and 15 lbs pressure for 30 minutes and kept at 55^0^C water bath for at least 30 min. t was then transferred to the anaerobic chamber and further supplemented with 1 ml/L of hemin solution (0.5mg/ml), 10 ml of ATCC vitamin mixture (ATCC, USA), 10 ml of ATCC mineral mixture (ATCC, USA) and 1.7 ml of acetic acid (30mM), 2 ml of butyric acid (4mM), 2 ml of propionic acid (8mM) and 100µl of iso-valeric acid (1mM).

For preparing media different antibiotic selection, mBHI was supplemented with antibiotics as per Minimum inhibitory concentration (MIC) data from EUCAST (*https://mic.eucast.org/Eucast2/SearchController/search.jsp?action=init*). The details of antibiotics used is given in Supplemental methods Table 1. To isolate spore-forming bacteria, fecal sample was subjected to heat and chloroform treatment before platting on to mBHI agar. For heat treatment, the sample was heat-treated at 70^0^C for 15 min and serially diluted before plating on mBHI agar. For chloroform treatment, fecal sample was incubated with 3% chloroform for an hour, serially diluted, and plated on mBHI agar. For all antibiotic treatments, 100 µl of the pooled fecal sample was serially diluted to 10^-5^ and 100 µl from the 10^-3^, 10^-4^ and 10^-5^ dilutions were plated on mBHI agar plates and were then incubated anaerobically using Coy Laboratories^®^ (85% nitrogen, 10% carbon-dioxide and 5% hydrogen) at 37^0^C for 96 hours. To isolate both the fast-growing and slow-growing bacteria, 50 colonies (Total CFU: 150) were picked after 48, 72 and 96 hours from plates based on the colony morphologies for each antibiotic supplement

*Identification of the bacteria:* A total of 1590 colnies were analyzed initially using MALDI-TOF (MALDI Biotyper, Bruker Inc) against reference spectra. Colonies that could not be identified with high confidence MALDI-TOF scores (≥2.0) against reference spectra were further identified using 16S rRNA gene sequencing. For 16S rDNA sequencing, total genomic DNA was prepared from the overnight culture of the strains using OMEGA E.Z.N.A genomic DNA isolation kit according to the manufacturer’s protocol. Quantity of DNA was measured using Qubit® 3.0 (Thermo Fisher Scientific Inc., MA) fluorometer and stored at -20^0^C until further use. Full length bacterial 16S rDNA was amplified using the universal forward and reverse primers 5’-AGA GTT TGA TCM TGG CTC AG-3’ (27F) and 5’- AAG GAG GTG ATC CAN CCR CA-3’ (1492R), respectively. For amplification of rDNA, 2X Dream Taq PCR mix (Cat: K1071, Thermofisher Scientific) was used for a final volume of 25 µl which consists of 1 µl of forward primer (10 nM), 1 µl of reverse primer (10 nM), 7.5 µl of DNAse/RNAse free water, 3 µl of genomic DNA (<10 ng/µl) and 12.5 µl of 2X PCR mix. Thermocycling conditions were used as followed, initial denaturation at 95^0^C for 2 mins, followed by 35 cycles of 95^0^C for 30 sec for denaturation, at 56^0^C for 30 sec for primer annealing, and for elongation at 72^0^C for 90 mins. The final elongation was at 72^0^C for 7 minutes. PCR products were then run in 1% agarose (Sigma) gel and the band of expected length was eluted using according to the manufacturer’s protocol. Eluted samples were quantified using Qubit and kept at -20^0^C until further use. The amplified DNA was subjected to Sanger sequencing initially using the 27F universal forward primer. In few cases primers 533F (GTGCCAGCMGCC GCGGTAA), 785F (GGATTAGATACCCTGGTA), and 805R (GACTACCAGGGTATCTAATC) and 1492R were used to obtain full length 16 s rRNA sequence.

*Fecal DNA isolation library preparation for Metagenomics sequencing :* Total community DNA from 0.25 g of fecal samples was extracted using the MoBio Powersoil DNA isolation kit according to the manufacturer’s instructions. Briefly, 0.25 g of fecal sample was added to the tubes with beads along with 60 µl of C1 solution and bead-beated for 10 min, centrifuged at 8000×g for 30 seconds and 400 µl of supernatant was transferred to a sterile microcentrifuge tube. The solution was treated with C2 and C3 solutions and DNA was precipitated using C4. Then, the solution was passed through spin column and washed once with ethanol before eluting with 30 µl of nuclease-free water. Quantity of DNA was measured using Qubit and was stored at -20^0^C until further use. High quality DNA after enrichment was used for shotgun metagenome sequencing while DNA from pure cultures was used for whole-genome sequencing. An initial DNA concentration of 0.3ng/ul was used for library preparation using Nextera XT library preparation kit (Illumina inc. San Diego, CA). Bead-based normalization was carried out before multiplexing of the samples in each run and sequencing was performed in Illumina Miseq platform (Illumina Inc., CA) using 2 x 250 or 2 x 300 base paired-end chemistry.

*Denovo assembly of genomes of isolates, error removal and ORF prediction:* Similar to metagenomic reads, Illumina sequencing reads of isolated genomes were first filtered for quality and sequencing adaptors with Prinseq (1). Filtered reads were then assembled *denovo* using Unicycler (2) with default parameters. Although Unicycler provides the best result for hybrid assembly with both short and long reads, our previous analyses showed that for Illumina reads, Unicycler resolves dead ends better when compared to Spades by reducing the low-confidence repeats mainly by end polishing using Pilon, Bowtie2, and Samtools. Each assembly result was checked individually with QUAST (3) and Bandage (4) for number of dead ends, N_50_ and GC peaks present. Gene calling for isolate genomes was done using Prokka (5).

*Preprocessing of metagenomics reads:* Low-quality reads were first filtered out from the metagenomics read sets using Prinseq (1). Sequencing adaptors and low-quality bases were removed from the reads for this purpose. After quality correction, human host read contaminations were removed from the data sets using Bowtie2 v.1.1.2 (9). Reference data from Human Genome Resource NCBI (https://www.ncbi.nlm.nih.gov/genome/guide/human/) was used as the reference for host read filtering. Bowtie2-build was used to index the human reference genome and filtered reads from the previous step were mapped against the database to eliminate human sequences. Unmapped reads were extracted from the resulted mapping files and saved for downstream analysis.

*Taxonomy assignments of the raw reads:* Taxonomic assignment of the metagenomics reads, obtained after host read removal, were done using classifier Kaiju (10). Reads were searched against the proGenomes (11) reference database of protein sequences that contain a non-redundant set from more than 25,000 genomes recovered by specI (12). NCBI-non-redundant database was also used for comparative analysis but has not been implemented for all the metagenomes due to high memory usage.

*Microbial diversity calculation from the metagenomic raw reads:* Simpson Dominance Index (D), Shannon Diversity Index (H) and Shannon Equitability Index (E_H_) was calculated by the following formulae;

$D= \sum_{i=1}^{s} {(\frac{n_{i}}{n})}^{2}= \sum{p_{i}}^{2}$, $H= - \sum_{i=1}^{s} p_{i}\mathrm{Ln}p_{i}$and $E_{H}=\frac{H}{H_{max}}=\frac{H}{Ln S}$

where, p_i_ = n_i_/n, n_i_=frequency of the i^th^ phylum for any sample and n=total frequency of all phylum of the same sample, S=total number of taxa for the same sample, H_max_=LnS

*Metagenomic assembly, contig correction, and ORF prediction:* Along with taxonomy assignment of the raw reads, unmapped reads afterhost read filtering step were assembled *denovo* using metaSPAdes (13) which is specifically designed for assembly of complex metagenomic communities. For initial assembly, reads were error corrected using spades-hammer using default parameters with k-mer size of 21, 33, 55, 77, 99 and 127. Assembly results were then checked with MetaQUAST (14) for error-prone assemblies. Contigs of less than 500 bp were removed from the resultant datasets. ORF predictions on the filtered contigs were then done with MetaGeneMark (15) with minimum length cutoff of 100 bp.

*Construction of non-redundant gene catalog and comparative mapping with existing gene catalog (IGC):* To generate the non-redundant gene set using both culture library and metagenomic datasets, cd-hit was used (16, 17). After gene calling, the concatenated datasets from the culture library were clustered using CD-hit at >95% identity with 90% overlap level. CD-hit was used with same criterion to create the non-redundant gene set from the metagenomic sequencing as well. These datasets were also checked using BLAT to avoid over-representation in the gene catalog. Finally, we mapped metagenomic as well as isolated genomes individually with previously published gene catalog (18) to check percentage recovery of isolates. CD-hit was used for this purpose with the previously mentioned parameters (18).

*Creation of population genome bins from metagenomic samples:* We used MaxBin2 (19) to generate population genome bins from the assembled reads of the metagenomes. To gain a better insight about the putative and hypothetical population genomes that could be present within the communities but could not be isolated, creating population genome bins is considered as a better alternative than taxonomy assignment of the raw reads. For this purpose, raw reads were mapped back on the assembled contigs using Bowtie2 (9) to get the coverage information. MaxBin2 relies on the differential coverage and GC values to create population genome bins. Following the generation of bins, we curated the total number of bins with >90% completeness and <5% redundancy for further downstream analysis. All the high confidence bins were further analyzed with specI (12) for species cluster determination. specI results were carefully considered for assignment of taxa for a particular bin based on ≥20 COGs found for every high-quality bin. As we have limited the output to species level only, those bins showing hits against multiple clusters were also selected, provided all the cluster belongs to the same species, where subspecies may vary.

*Coverage estimation for isolates, low vs. high abundant taxa:* To determine the abundance of each isolated species in the pooled samples, we measured the coverage by read mapping with Bowtie2 (9) at 95% identity level. To obtain proper representation from the pooled sample, we used high depth sequencing coverage for the pooled sample.

*Comparative metagenomics:* KEGG annotation for the ORFs was obtained for pooled (S7) sample metagenome and a mixture of 102 isolates along with the strains that inhibit R20291. For the isolates, ORFs from individual species were mixed to create a combination of pooled set. Finally, all four datasets were searched for KO modules using GhostKOALA (20). Heat-map using hierarchical clustering of the datasets generated with R (<http://www.R-project.org/>).

*Clostridium difficile selective media and enumeration of C. difficile in co-culture assays: C. difficile* agar (Acumedia Manufacturers Inc.) was used for preparing the base medium according to manufacturer’s recommendation. Antibiotics D-cycloserine and cefoxitin were added to the base medium to make the final concentration at 0.5 g/L and 16 mg/L respectively. The prepared *C. difficile* selective medium (CDSA) was then kept at 4^0^C until use.

*Statistics:* Statistical analysis of the data was performed using GraphPad Prism V 8.0. For the Principal Component Analysis (PCA), “ggfortify” (https://CRAN.R-project.org/package=ggfortify) from the R-package was used. Heat-map using hierarchical clustering of the datasets was done using heatmap.2 function within gplots package in R (<https://CRAN.R-project.org/package=gplots>).

**Supplemental methods Table 1:** Media composition and culture conditions used for culturomics

| **Composition of modified Brain Heart Infusion broth (mBHI)** | |
| --- | --- |
| **Ingredients** | **per liter** |
| Brain heart infusion (BHI) | 37.0 g |
| Yeast extract | 5.0 g |
| L cysteine | 0.3 g |
| Resazurine (0.25 mg/ml solution) | 1 ml |
| Agar | 15.0 g |
| Menadione (5.8 M solution) | 1 ml |
| Vitamin mix ATCC | 10 ml |
| Mineral mix ATCC | 10 ml |
| Hemin (0.5mg/ml solution)) | 1 ml |
| Acetic acid (30mM solution) | 1.7 ml |
| Propionic acid (8mM solution) | 2 ml |
| Isovaleric acid (1mM solution) | 100 µl |
| Butyric acid (4mM solution) | 2 ml |
| **Culture conditions used** |  |
| Culture condition 1 | mBHI alone |
| Culture condition 2 | mBHI + 1 ug/ml Sulphamethoxazole |
| Culture condition 3 | mBHI + 0.5 ug/ml Ciprofloxacin |
| Culture condition 4 | mBHI + 2 ug/ml Erythromycin |
| Culture condition 5 | mBHI + 0.06ug/ml Chlortetracycline |
| Culture condition 6 | mBHI + 2.0 ug/ml Erythromycin + 0.5 ug/ml Ciprofloxacin |
| Culture condition 7 | mBHI + 0.5 ug/ml Imepenem |
| Culture condition 8 | mBHI+ 1 ug/ml Gentamycin |
| Culture condition 9 | mBHI + 0.5 ug/ml Vancomycin |
| Culture condition 10 | mBHI + 1.0 1.0 µg/ml Aztreonam + 10.ug/ml Colistin sulphate + 2.0 ug/ml Gentamycin + 0.5ug/ml Ampicillin + 2.0ug/ml Erythromycin and 0.25ug/ml vancomycin |
| Culture condition 11 | heating samples at 70 ^O^C for 15 minutes and plating on mBHI |
| Culture condition 12 | treating sample with 3% chloroform for 1 hour and plating on mBHI |

**Supplemental References**

1. Schmieder R, Edwards R. 2011. Quality control and preprocessing of metagenomic datasets. Bioinformatics 27:863-4.

2. Wick RR, Judd LM, Gorrie CL, Holt KE. 2017. Unicycler: Resolving bacterial genome assemblies from short and long sequencing reads. PLoS Comput Biol 13:e1005595.

3. Gurevich A, Saveliev V, Vyahhi N, Tesler G. 2013. QUAST: quality assessment tool for genome assemblies. Bioinformatics 29:1072-5.

4. Wick RR, Schultz MB, Zobel J, Holt KE. 2015. Bandage: interactive visualization of de novo genome assemblies. Bioinformatics 31:3350-2.

5. Seemann T. 2014. Prokka: rapid prokaryotic genome annotation. Bioinformatics 30:2068-9.

6. Wajid B, Serpedin E. 2016. Do it yourself guide to genome assembly. Brief Funct Genomics 15:1-9.

7. Larkin MA, Blackshields G, Brown NP, Chenna R, McGettigan PA, McWilliam H, Valentin F, Wallace IM, Wilm A, Lopez R, Thompson JD, Gibson TJ, Higgins DG. 2007. Clustal W and Clustal X version 2.0. Bioinformatics 23:2947-8.

8. Tamura K, Stecher G, Peterson D, Filipski A, Kumar S. 2013. MEGA6: Molecular Evolutionary Genetics Analysis version 6.0. Mol Biol Evol 30:2725-9.

9. Langmead B, Trapnell C, Pop M, Salzberg SL. 2009. Ultrafast and memory-efficient alignment of short DNA sequences to the human genome. Genome Biol 10:R25.

10. Menzel P, Ng KL, Krogh A. 2016. Fast and sensitive taxonomic classification for metagenomics with Kaiju. Nat Commun 7:11257.

11. Mende DR, Letunic I, Huerta-Cepas J, Li SS, Forslund K, Sunagawa S, Bork P. 2017. proGenomes: a resource for consistent functional and taxonomic annotations of prokaryotic genomes. Nucleic Acids Res 45:D529-D534.

12. Mende DR, Sunagawa S, Zeller G, Bork P. 2013. Accurate and universal delineation of prokaryotic species. Nat Methods 10:881-4.

13. Nurk S, Meleshko D, Korobeynikov A, Pevzner PA. 2017. metaSPAdes: a new versatile metagenomic assembler. Genome Res 27:824-834.

14. Mikheenko A, Saveliev V, Gurevich A. 2016. MetaQUAST: evaluation of metagenome assemblies. Bioinformatics 32:1088-90.

15. Zhu W, Lomsadze A, Borodovsky M. 2010. Ab initio gene identification in metagenomic sequences. Nucleic Acids Res 38:e132.

16. Fu L, Niu B, Zhu Z, Wu S, Li W. 2012. CD-HIT: accelerated for clustering the next-generation sequencing data. Bioinformatics 28:3150-2.

17. Li W, Godzik A. 2006. Cd-hit: a fast program for clustering and comparing large sets of protein or nucleotide sequences. Bioinformatics 22:1658-9.

18. Li J, Jia H, Cai X, Zhong H, Feng Q, Sunagawa S, Arumugam M, Kultima JR, Prifti E, Nielsen T, Juncker AS, Manichanh C, Chen B, Zhang W, Levenez F, Wang J, Xu X, Xiao L, Liang S, Zhang D, Zhang Z, Chen W, Zhao H, Al-Aama JY, Edris S, Yang H, Wang J, Hansen T, Nielsen HB, Brunak S, Kristiansen K, Guarner F, Pedersen O, Dore J, Ehrlich SD, Meta HITC, Bork P, Wang J, Meta HITC. 2014. An integrated catalog of reference genes in the human gut microbiome. Nat Biotechnol 32:834-41.

19. Wu YW, Simmons BA, Singer SW. 2016. MaxBin 2.0: an automated binning algorithm to recover genomes from multiple metagenomic datasets. Bioinformatics 32:605-7.

20. Kanehisa M, Sato Y, Morishima K. 2016. BlastKOALA and GhostKOALA: KEGG Tools for Functional Characterization of Genome and Metagenome Sequences. J Mol Biol 428:726-731.
