# Supplementary figures and images for: Identification of Clostridioides difficile-Inhibiting Gut Commensals Using Culturomics, Phenotyping, and Combinatorial Community Assembly
Source: mSystems. 2020 Feb 4;5(1):e00620-19. doi: 10.1128/mSystems.00620-19 (PMC7002114; doi:10.1128/mSystems.00620-19)

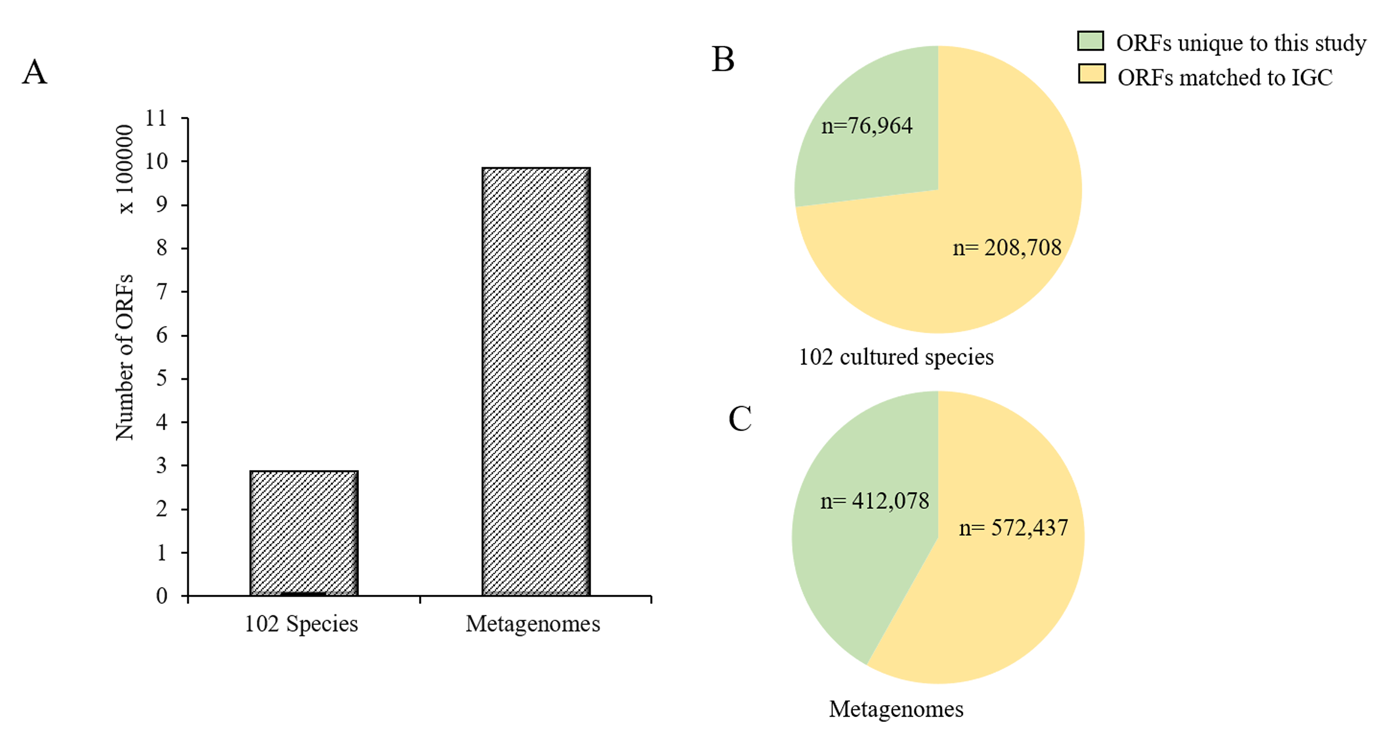

Supplement: FIG S1 [file mSystems.00620-19-sf001.tif]

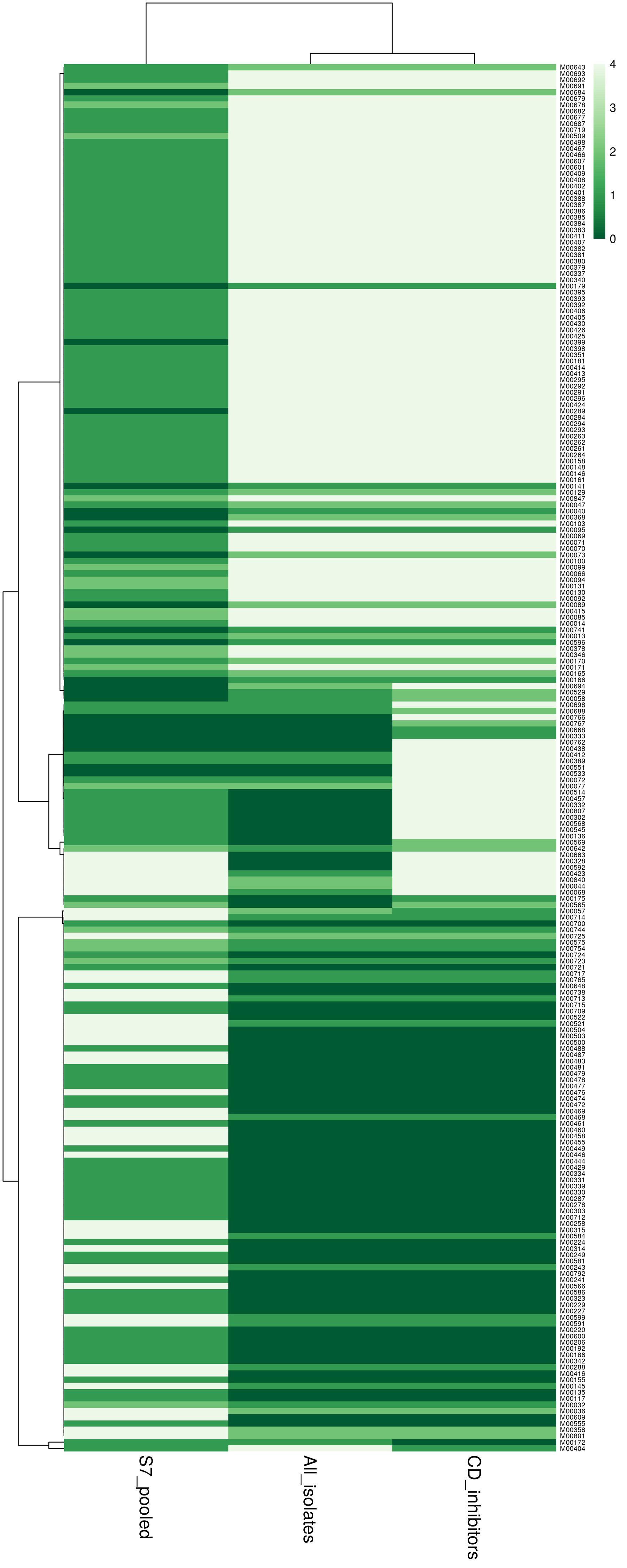

Supplement: FIG S2 [file mSystems.00620-19-sf002.jpg]
